# Foxp3-Positive Regulatory T Cells Contribute to Antifibrotic Effects in Renal Fibrosis via an Interleukin-18 Receptor Signaling Pathway

**DOI:** 10.3389/fmed.2020.604656

**Published:** 2020-12-02

**Authors:** Yasuaki Hirooka, Yuji Nozaki, Kaoru Niki, Asuka Inoue, Masafumi Sugiyama, Koji Kinoshita, Masanori Funauchi, Itaru Matsumura

**Affiliations:** ^1^Department of Rheumatology, Kindai University Nara Hospital, Nara, Japan; ^2^Department of Hematology and Rheumatology, Kindai University School of Medicine, Osaka, Japan

**Keywords:** IL-18, IL-18 receptor, renal fibrosis, unilateral ureteral obstruction, regulatory T cells

## Abstract

Renal interstitial fibrosis is a common lesion in the process of various progressive renal diseases. Interleukin (IL)-18 is a proinflammatory cytokine that plays an important role in the induction of Th1 responses and is associated with renal interstitial fibrosis, but the mechanism of fibrosis remains unclear. Here we used IL-18 receptor alpha knockout (IL-18Rα KO) mice to investigate the role of an IL-18Rα signaling pathway in renal fibrosis in a murine model of unilateral ureteral obstruction. IL-18 Rα KO mice showed decreased renal interstitial fibrosis and increased infiltration of CD4+ T cells and Foxp3+ regulatory T cells (Tregs) compared to wildtype (WT) mice. The expression of renal transforming growth factor beta 1 (TGF-β1, which is considered an important cytokine in renal interstitial fibrosis) was not significantly different between WT and IL-18Rα KO mice. The adoptive transfer of CD4+ T cells from the splenocytes of IL-18Rα KO mice to WT mice reduced renal interstitial fibrosis and increased the number of Foxp3+ Tregs in WT mice. These results demonstrated that Foxp3+ Tregs have a protective effect in renal interstitial fibrosis via an IL-18R signaling pathway.

## Introduction

Renal interstitial fibrosis is a common and important lesion in the process of various progressive renal diseases that progress to renal atrophy. The pathological features of renal interstitial fibrosis are renal tubular atrophy, a reduction or disappearance of peritubular capillaries, and an increase in the extracellular matrix (ECM) (1–3). Interleukin (IL)-18 is a proinflammatory cytokine and member of the IL-1 family, and it is produced by macrophages, dendritic cells, epithelial cells, keratinocytes, and other cell types (4–6). IL-18 is stored intracellularly as a biologically inactive precursor (pro-IL-18), similar to IL-1β, and is secreted extracellularly as the bioactive mature form of IL-18 after being cleaved by IL-1β-converting enzyme (caspase 1). IL-18 recognizes a heterodimeric receptor, which consists of unique α [IL-1 receptor(R)-related protein] signaling chain and non-binding β (IL-1R-accessory protein-like) signaling chain (7). IL-18 promotes the production of interferon gamma (IFN-γ) and strongly induces a Th1 response (8).

In the kidney, IL-18 is expressed in the renal tubular epithelium. Patients with chronic kidney disease or nephrotic syndrome exhibit elevated levels of IL-18 (9–12). IL-18 is associated with renal interstitial fibrosis, and IL-18 neutralization has been shown to prevent renal injury and fibrosis in unilateral ureteral obstruction (UUO) mice (13). However, the mechanism of IL-18 during renal obstruction remains unclear. We conducted the present study to: (1) determine whether renal interstitial fibrosis is reduced in IL-18Rα knockout (KO) mice undergoing UUO, and (2) elucidate the mechanisms underlying fibrosis.

## Materials and Methods

### Ethics Statement

The animal protocols were approved by the Kindai University Animal Care Committee and were performed in accordance with the Kindai University Animal Care Guidelines (KAME-22-014, 1/4/2010).

### Animals

IL-18Rα-deficient mice on a C57BL/6 background were kindly provided by Dr. Shizuo Akira (Osaka University, Osaka, Japan). The C57BL/6 mice used as the wildtype control (WT) were purchased from the Shizuoka Laboratory Animal Center (Shizuoka, Japan). All mice were bred in our specific pathogen-free animal facility. Eight-week-old female mice were used in this study.

### UUO Protocol

For the UUO model, the mouse was anesthetized with inhaled isoflurane and underwent left ureteral obstruction (the controls underwent a sham operation). The left ureter was isolated and completely ligated with a 3–0 silk suture. Sham-operated animals underwent the same surgery without ureteral ligation. Operated mice were re-anesthetized and culled at day 3 (WT and IL-18RαKO; *n* = 6 and 5), day 7 (*n* = 5), or day 14 (*n* = 6) after surgery. The left kidneys were harvested for analysis. Blood was collected in heparinized tubes for the measurement of blood urea nitrogen (BUN) and IL-18.

### Histological Analysis

Kidney tissues were fixed in 10% buffered formalin, embedded in paraffin, sectioned, and stained with periodic acid-Schiff-stained (PAS) reagent. Tubular injury was evaluated based on a semiquantitative scale by determining the percentage of cortical tubules in which epithelial necrosis, loss of brush border, cast formation, and tubular dilation were evaluated: 0 = normal kidney; 1 = 1–25%; 2 = 26–50%; 3 = 51–75%; 4 = 76–100% tubules injured.

CD4+ T cells, kidney injury molecule-1 (Kim-1), and type IV collagen were demonstrated by the immunoperoxidase staining of frozen 6-μm-thick periodate-lysine-paraformaldehyde-fixed kidney sections, as described (14). F4/80+ cells as macrophages, α-smooth muscle actin (α-SMA) and Forkhead box protein 3 (Foxp3) as regulatory T cells (Tregs), and cleaved caspase-3 were identified in 4-μm-thick formalin-fixed sections as described (14, 15). The numbers of CD4+ T cells, F4/80+ cells, Foxp3+ cells, and cleaved caspase-3+ cells were assessed in 10 fields per slide at ×400 magnification, and the results are expressed as cells per high-power field (c/hpf). Tubular Kim-1 immunostaining was quantified by counting the number of positively stained tubules in 10 fields per slide at a magnification of ×400. A positive tubule cross-section was defined as having two or more stained cells. The positive area of type IV collagen and α-SMA were assessed in 10 fields per slide at ×400 magnification with a fluorescence microscope and analyzer (model BZ-X700, Keyence, Osaka, Japan).

The primary monoclonal antibodies used were rat monoclonal antibody GK1.5 for CD4+ T cells (Pharmingen, San Diego, CA), F4/80 hybridoma culture supernatant (HB198; American Type Culture Collection, Manassas, MD), rat monoclonal antibody for TIM-1 (R&D Systems, Minneapolis, MN), mouse monoclonal antibody for α-SMA (Sigma-Aldrich, St. Louis, MO), rabbit polyclonal antibody for type IV collagen (ab6586; Abcam, Cambridge, United Kingdom), mouse/rat monoclonal antibody for Foxp3 (FJK-16s; eBioscience, Hatfield, United Kingdom), and rabbit antibody recognizing the cleaved form of caspase-3 (Cell Signaling Technology, Beverly, MA).

### Real-Time PCR Analysis

We performed a real-time polymerase chain reaction (PCR) as described (14) for the measurement of the intrarenal mRNA expressions of IFN-γ, monocyte chemoattractant protein-1 (MCP-1/CCL2), matrix metalloproteinase-2 (MMP-2), Foxp3, and 18SrRNA by using FastStart DNA master Sybr Green I (Applied Biosystems, Foster City, CA) and the expressions of IL-6, IL-10, IL-12, IL-18R, tumor necrosis factor-alpha (TNF-α), transforming growth factor-β1 (TGF-β1), Kim-1, and 18S RNA by using Taqman gene (Applied Biosystems) on whole kidney tissue. The sequences of the primers and the gene database number are listed in Tables 1, 2. The relative amount of mRNA was calculated using the comparative Ct (ΔΔCt) method. All specific amplicons were normalized against 18SrRNA, which was amplified in the same reaction as an internal control using commercial reagents (Applied Biosystems) and is expressed as the fold increase relative to the data of the sham-operated mice.

**Table 1:** Primer sequences for analysis of mRNA expression.

	**Forward primer**	**Reverse primer**
18SrRNA	GTAACCCGTTGAACCCCATTC	GCCTCACTAAACCATCCAATCG
IFN-γ	TGCTGATGGGAGGAGATGTCT	TTTCTTTCAGGGACAGCCTGTT
MCP-1	AAAAACCTGGATCGGAACCAA	CGGGTCAACTTCACATTCAAAG
MMP-2	ACCCAGATGTGGCCAACTAC	GAGCAAAGGCATCATCCACT
Foxp3	GGCCCTTCTCCAGGACAGAC	TCCACAGTGGAGAGCTGATGC

*IL, interleukin; IFN, interferon; MCP, monocyte chemoattractant protein; MMP, matrix metalloproteinase; Foxp, Forkhead box protein*.

**Table 2:** Gene database number for analysis of mRNA expression.

	**Forward primer**
18SrRNA	NM_026744.3
IL-6	Mm00446190
IL-10	Mm99999062_m1
IL-12p40	Mm00434174_m1
IL-18R1 (IL-18Rα)	Mm00515180_m1
TNF-α	Mm99999068_m1
TGF-β1	Mm03024053_m1
Kim-1	Mm00506686_m1

*IL, interleukin; TNF, tumor necrosis factor; TGF, transforming growth factor; Kim, kidney injury molecule*.

### Serum IL-18 Quantitation by ELISA

The serum IL-18 levels in the mice were determined by an enzyme-linked immunosorbent assay (ELISA) kit (BD Biosciences, San Diego, CA) as described (14).

### Cell Sorting and Adoptive Transfer Experiments

CD4+ T cells were isolated using a BD FACSAria special-order research product (Becton Dickinson, Lincoln Park, NJ) for 90–95% purity from splenocytes in IL-18Rα KO mice for the adoptive transfer. Approximately 2 × 10^6^ CD4+ T cells were injected intravenously into WT mice 3 days before they were subjected to the UUO operation. WT mice that received a transfer of CD4+ T cells were sacrificed at day 7 after surgery (n = 10). WT mice transferred with CD4+ T cells were compared to non-transferred WT mice. The flow cytometry antibody was FITC-anti-CD4 (BD Bioscience). An isotype-matched irrelevant monoclonal antibody was used. Cells that fluoresced at levels above the negative control were considered positive.

### Statistical Analyses

The results are expressed as the mean ± SEM. Groups were compared by the Mann-Whitney U-test. Multiple comparisons were analyzed using Dunn's multiple comparisons test. We analyzed the data using GraphPad Prism software (GraphPad, La Jolla, CA). Differences were accepted as significant when the *p* < 0.05.

## Results

### Renal IL-18R Expression and Serum IL-18 Levels

**Figure 1:**
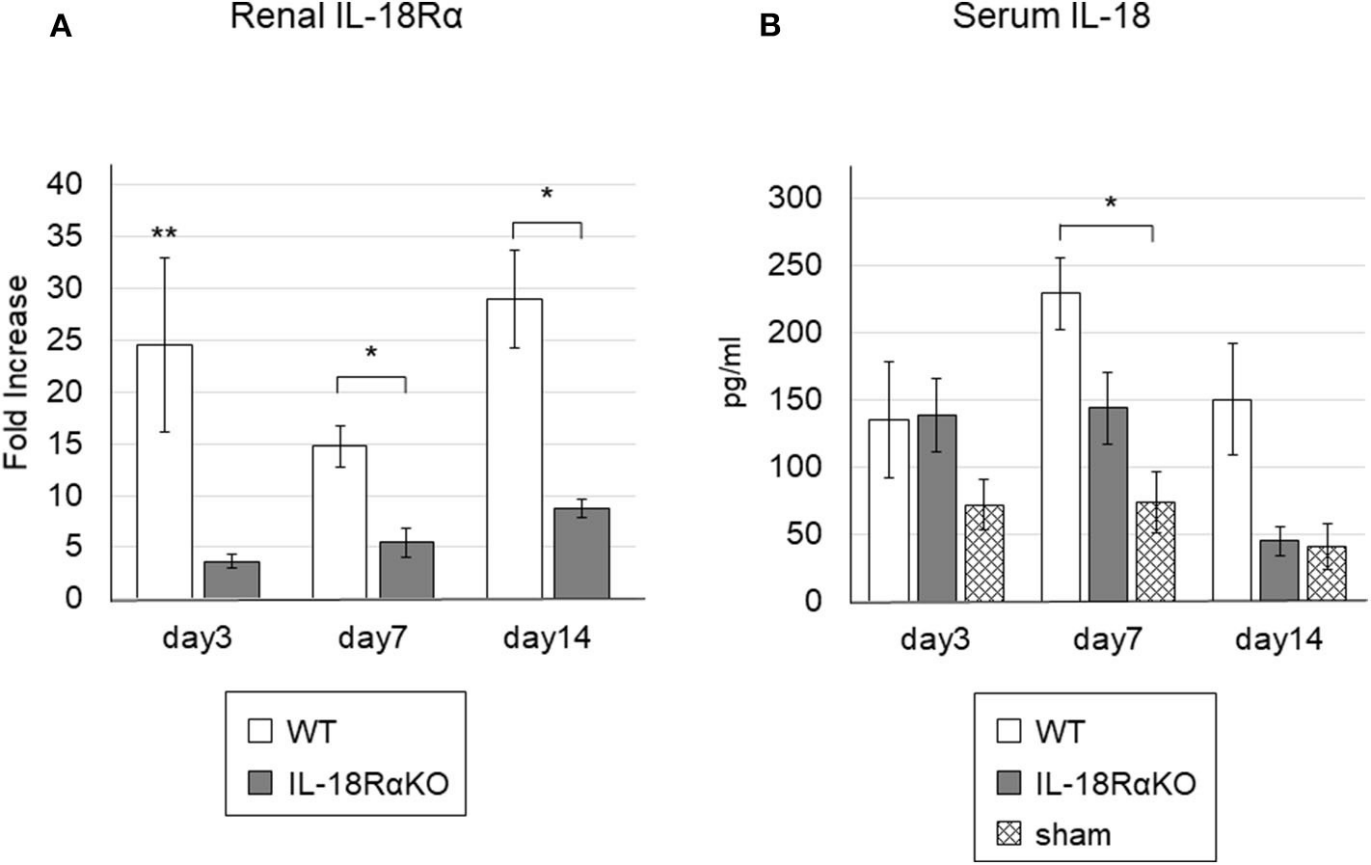
Gene expressions of IL-18Rα in the mouse kidney and the levels of serum IL-18. **(A)** Gene expressions of IL-18Rα measured by real-time PCR. **(B)** The levels of IL-18 in the blood. The data are mean ± SEM. *p < 0.05, **p < 0.01.

The renal IL-18Rα expression as measured by real-time PCR is shown in Figure 1A. The renal IL-18Rα expression in IL-18RαKO mice was significantly decreased on days 3, 7, and 14 compared to the expression in WT mice. Figure 1B illustrates the serum IL-18 levels measured by ELISA. The serum IL-18 levels in WT mice were significantly increased on day 7 compared to those in sham-operated mice. On day 14, IL-18RαKO mice tended to have lower serum IL-18 levels compared to WT mice, although the difference was not statistically significant (*P* = 0.07), and their serum IL-18 levels were similar to those of sham-operated mice.

### Functional and Structural Aggravation From UUO

**Figure 2:**
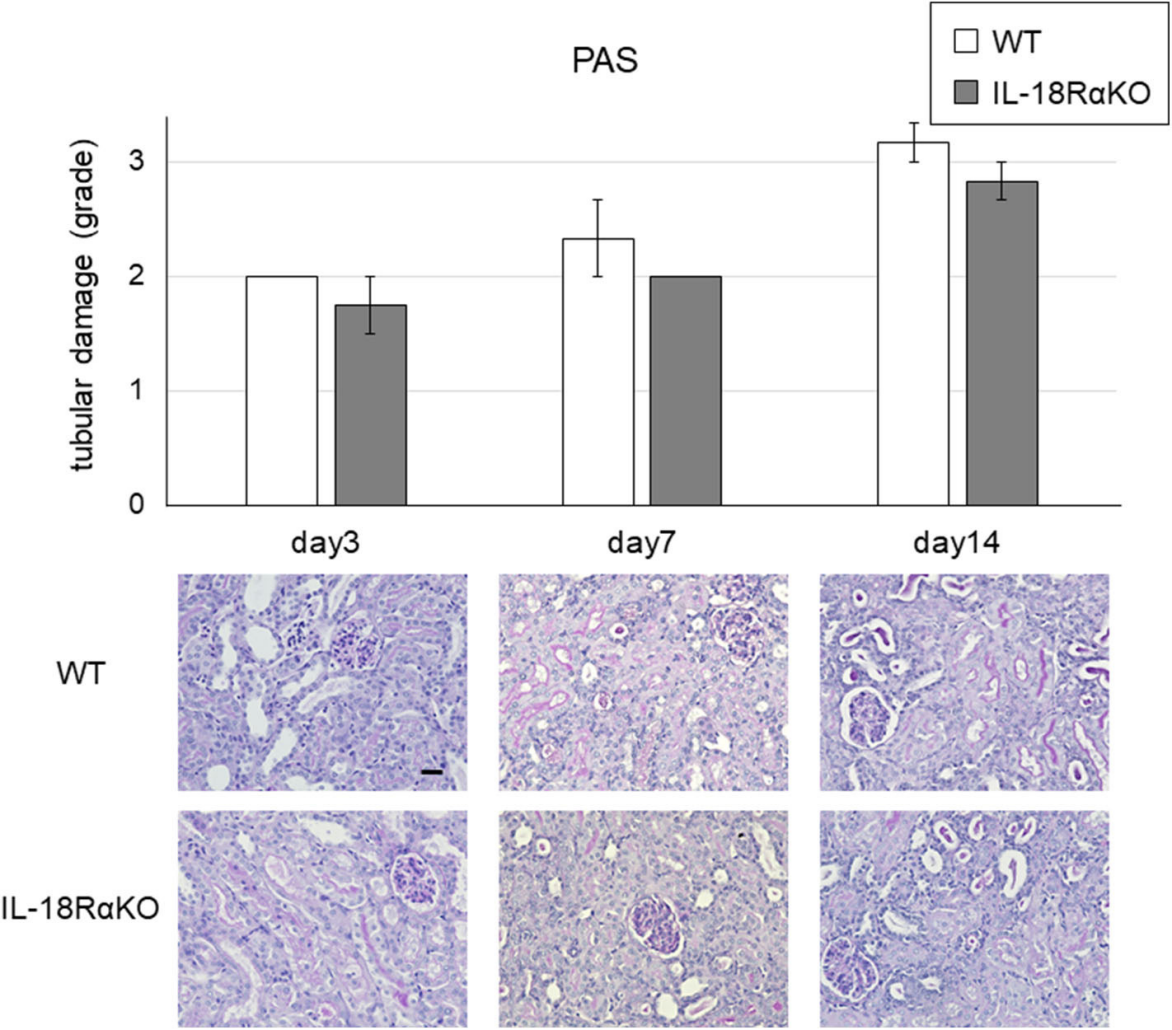
Representative light microscopy of periodic acid Schiff (PAS)-stained renal sections after unilateral ureteral obstruction (UUO). Tubular injury in the cortex of the kidney was scored (see the Materials and Methods section for the scoring method). The data are mean ± SEM. Original magnification ×400. Scale bar, 50 μm.

**Figure 3:**
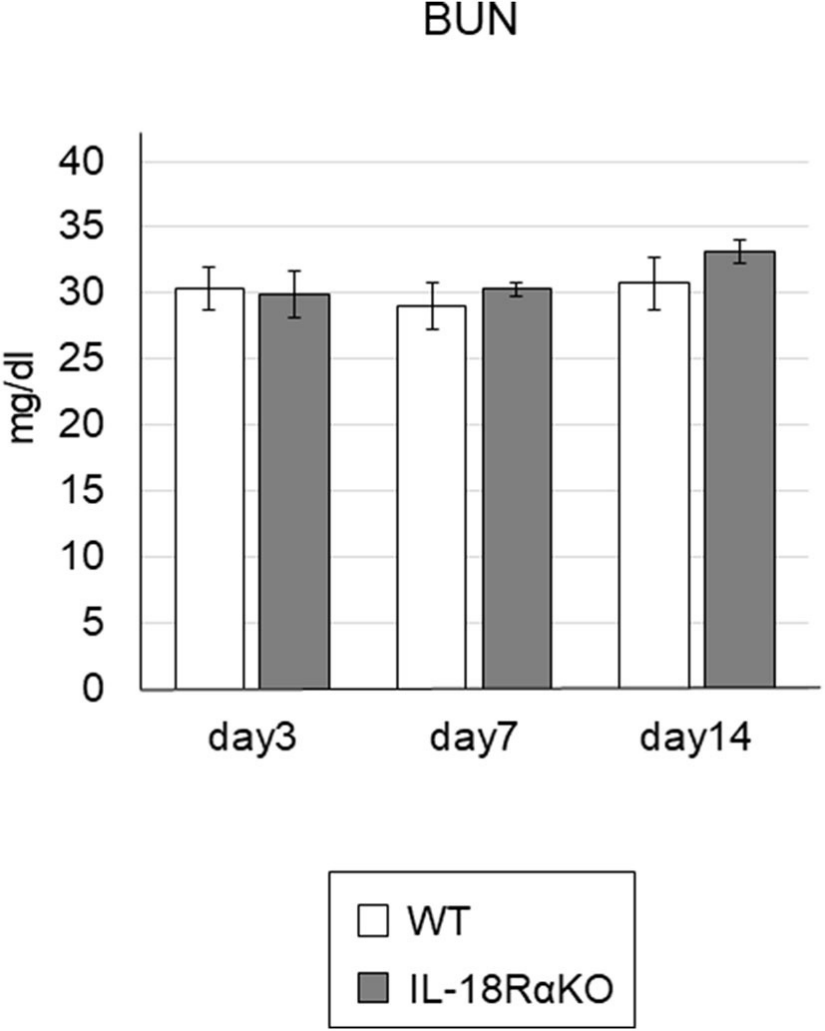
The levels of BUN. BUN was measured from blood samples as an indicator of kidney function. Data shown are the mean ± SEM.

The semiquantitative tubular injury scores obtained by PAS staining are shown in Figure 2. The intensity of tubular injury gradually increased from day 3 to day 14 after ureteral ligation. There was no significant difference in the tubular injury score between WT mice and IL-18RαKO mice. Figure 3 shows the BUN levels as an indications of kidney function. No significant difference in BUN levels was observed between WT and IL-18RαKO mice.

### Expression of Type IV Collagen

**Figure 4:**
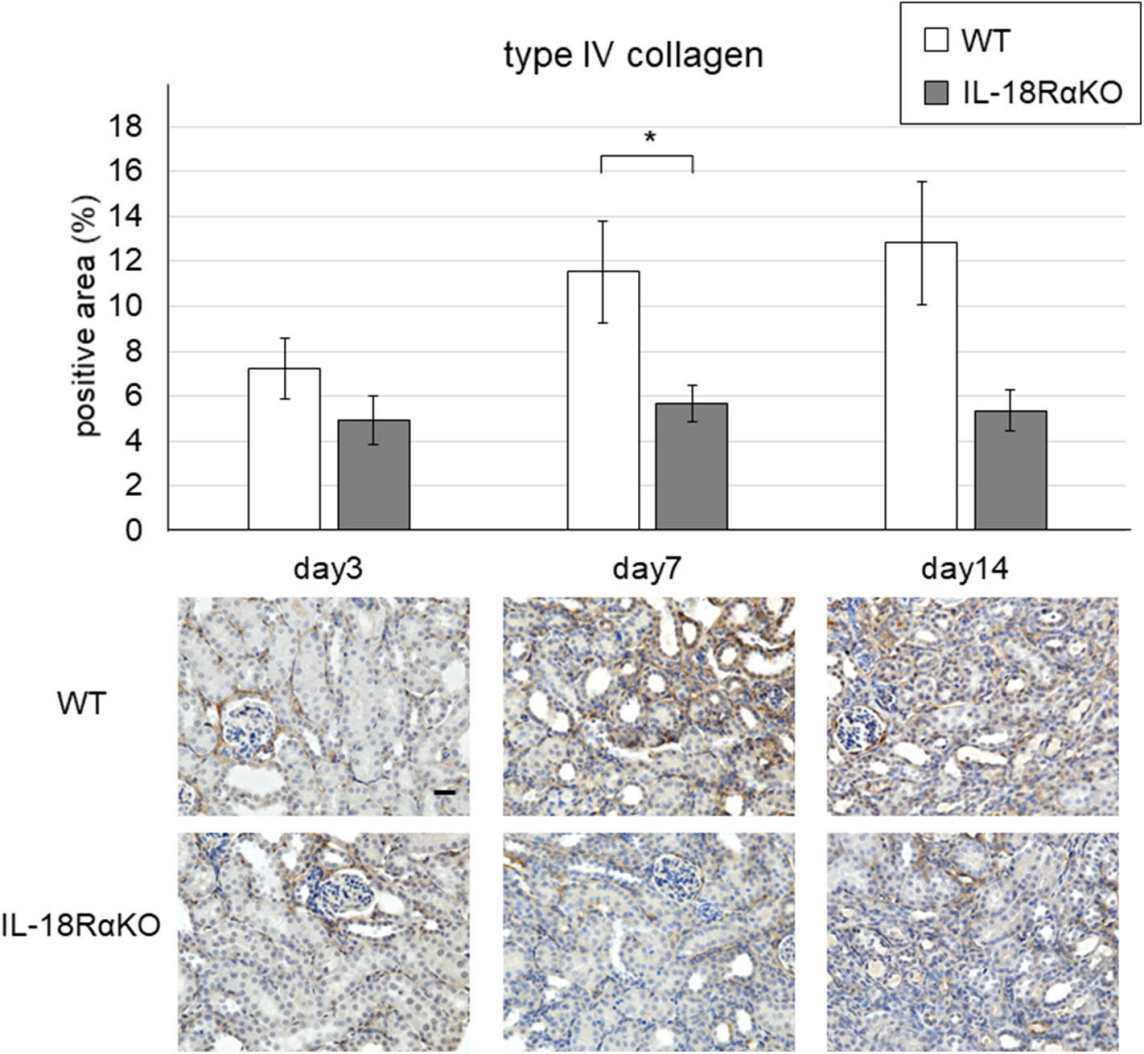
Expression of type IV collagen in the mouse kidney. The interstitial type IV collagen expression in IL-18RαKO mice was significantly reduced compared to that in WT mice on day 7 after unilateral ureteral obstruction. Data shown are the mean ± SEM. Original magnification ×400. Scale bar, 50 μm. *p < 0.05.

Figure 4 shows representative results of the immunohistochemical staining for type IV collagen. The interstitial type IV collagen expression in IL-18RαKO mice was significantly decreased on day 7 compared to that in WT mice. On day 14, IL-18Rα KO mice tended to have lower expression of type IV collagen compared to WT mice, although the difference was not statistically significant (*P* = 0.09).

### Expression of α-SMA

**Figure 5:**
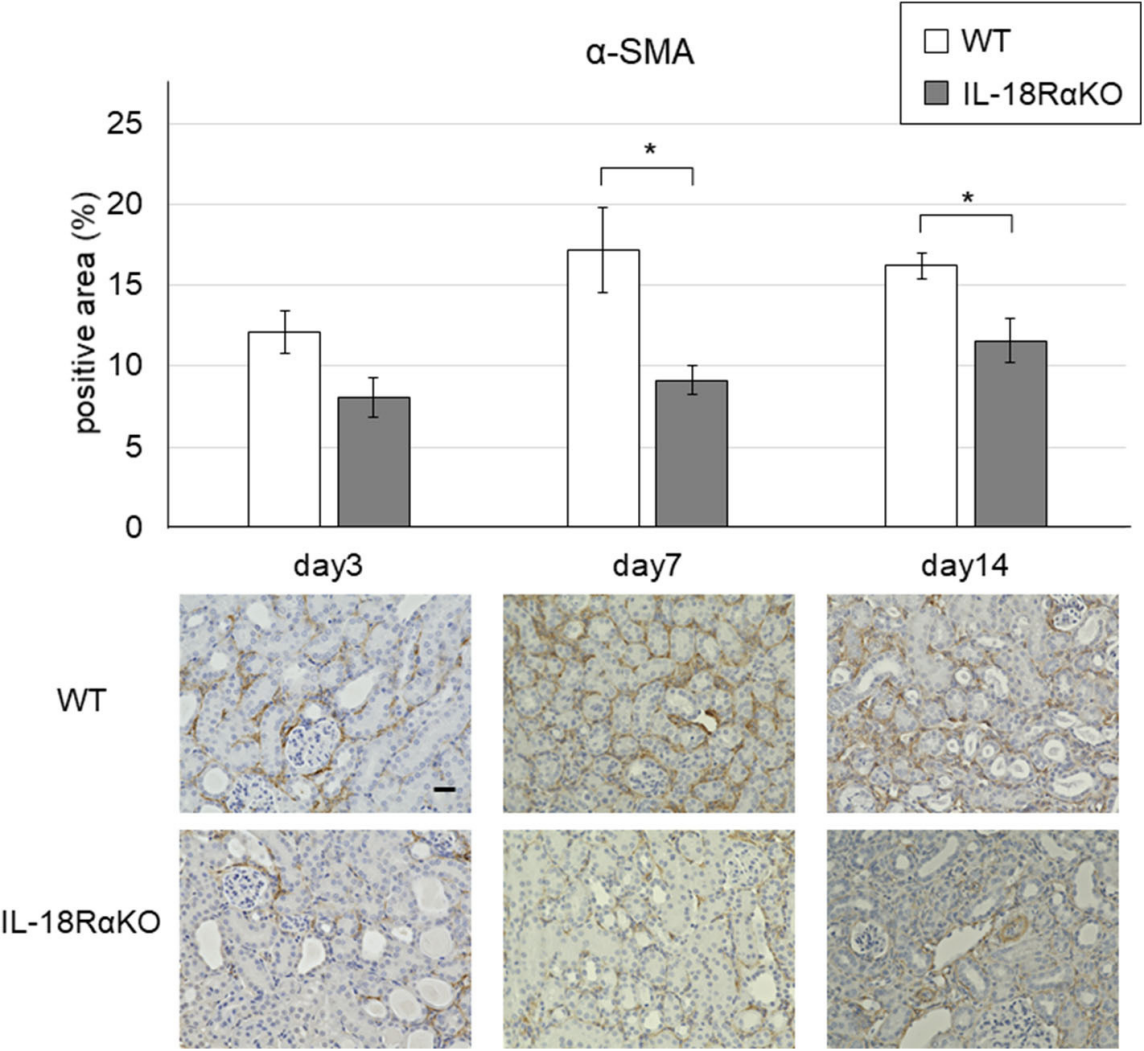
Expression of α-SMA in the mouse kidney. The interstitial α-SMA expression in IL-18RαKO mice was significantly reduced compared to WT mice on days 7 and 14 after unilateral ureteral obstruction. The data are mean ± SEM. Original magnification ×400. Scale bar, 50 μm. *p < 0.05.

Examples of the immunohistochemical staining for α-SMA are shown in Figure 5. The interstitial α-SMA expression gradually increased from day 3 to day 14 after ureteral ligation. On days 7 and 14, the interstitial α-SMA expression in IL-18RαKO mice was significantly reduced compared to that in WT mice.

### Expression of Kim-1

**Figure 6:**
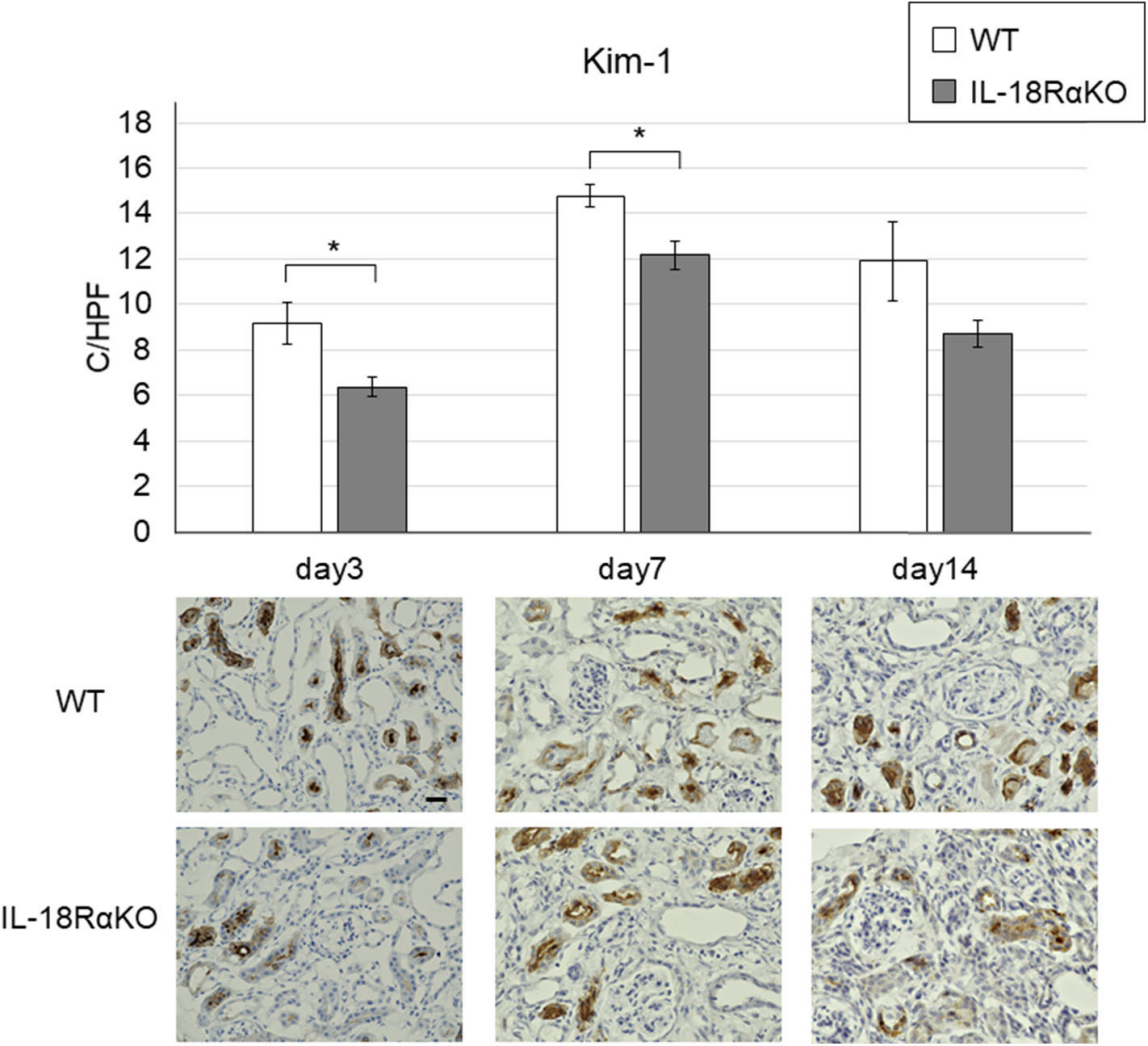
Expression of Kim-1 in the mouse kidney. Kim-1+ tubules were significantly decreased in IL-18RαKO mice on days 3,7, and 14 compared to WT mice. The data are mean ± SEM. Original magnification ×400. Scale bar, 50 μm. *p < 0.05.

Figure 6 provides examples of the immunohistochemical staining for tubular Kim-1. Kim-1+ tubules were not observed in the sham-operated mice, whereas the Kim-1 expression was increased in WT and IL-18RαKO mice. Kim-1+ tubules in IL-18RαKO mice were decreased significantly on days 3 and 7 compared to WT mice. We also investigated the Kim-1 mRNA expression as measured by real-time PCR and observed that the expression in IL-18RαKO mice was decreased significantly on days 7 and 14 compared to WT mice (Table 3).

**Table 3:** Expression of renal mRNA.

	**day3**	**day7**	**day14**
	**WT vs. IL-18RαKO**	**WT vs. IL-18RαKO**	**WT vs. IL-18RαKO**
IL-6	123.3 ± 31.36 vs. 93.4 ± 19.0	24.1 ± 4.3 vs. 26.8 ± 5.4	53.8 ± 5.7 vs. 47.0 ± 6.2
IL-10	82.9 ± 26.2 vs. 39.4 ± 11.5	34.7 ± 4.3 vs. 31.8 ± 5.4	31.4 ± 8.0 vs. 41.7 ± 10.0
IL-12	3.5 ± 0.7 vs. 4.0 ± 0.7	5.0 ± 0.5 vs. 6.0 ± 0.8	16.7 ± 1.8 vs. 25.3 ± 4.9
IFN-γ	4.2 ± 0.2 vs. 3.5 ± 0.7	9.1 ± 0.4 vs. 7.2 ± 0.6	12.2 ± 2.0 vs. 12.3 ± 3.0
TNF-α	18.1 ± 4.1 vs. 20.5 ± 5.7	12.4 ± 2.2 vs. 13.9 ± 2.2	22.1 ±2.6 vs. 23.0 ± 1.8
TGF-β1	9.1 ± 2.4 vs. 7.4 ± 1.4	5.1 ± 0.7 vs. 4.4 ± 0.6	7.8 ± 0.6 vs. 6.2 ± 0.8
MCP-1	29.3 ± 4.7 vs. 24.1 ± 5.5	30.5 ± 1.3 vs. 50.4 ± 8.6	35.2 ± 5.6 vs. 31.4 ± 3.2
MMP-2	8.1 ± 1.1 vs. 6.2 ± 1.0	41.1 ± 4.2 vs. 30.4 ± 5.3	30.1 ± 3.7 vs. 33.6 ± 6.4
Kim-1	2778.1 ± 595.3 vs. 1687.8 ± 334.7	385.7 ± 49.8 vs. 206.9 ± 25.5^**^	272.9 ± 47.4 vs. 137.5 ± 18.8^*^
Foxp3	1.36 ± 0.1 vs. 2.0 ± 0.1^**^	4.4 ± 0.4 vs. 8.7 ± 1.5^*^	12.4 ± 1.3 vs. 20.8 ± 3.0^*^

*Cytokines, chemokines, and fibrosis-related molecules were measured by real-time PCR in WT and IL-18RαKO mice on days 3, 7, and 14 after unilateral ureteral obstruction. In each experiment, the expression levels were normalized to the expression of 18SrRNA and are expressed relative to the values of sham-operated mice. The data are mean fold-increase ± SEM*.

* *p < 0.05*,

** *p < 0.01*.

### The Infiltration of CD4+ T Cells and Macrophages

**Figure 7:**
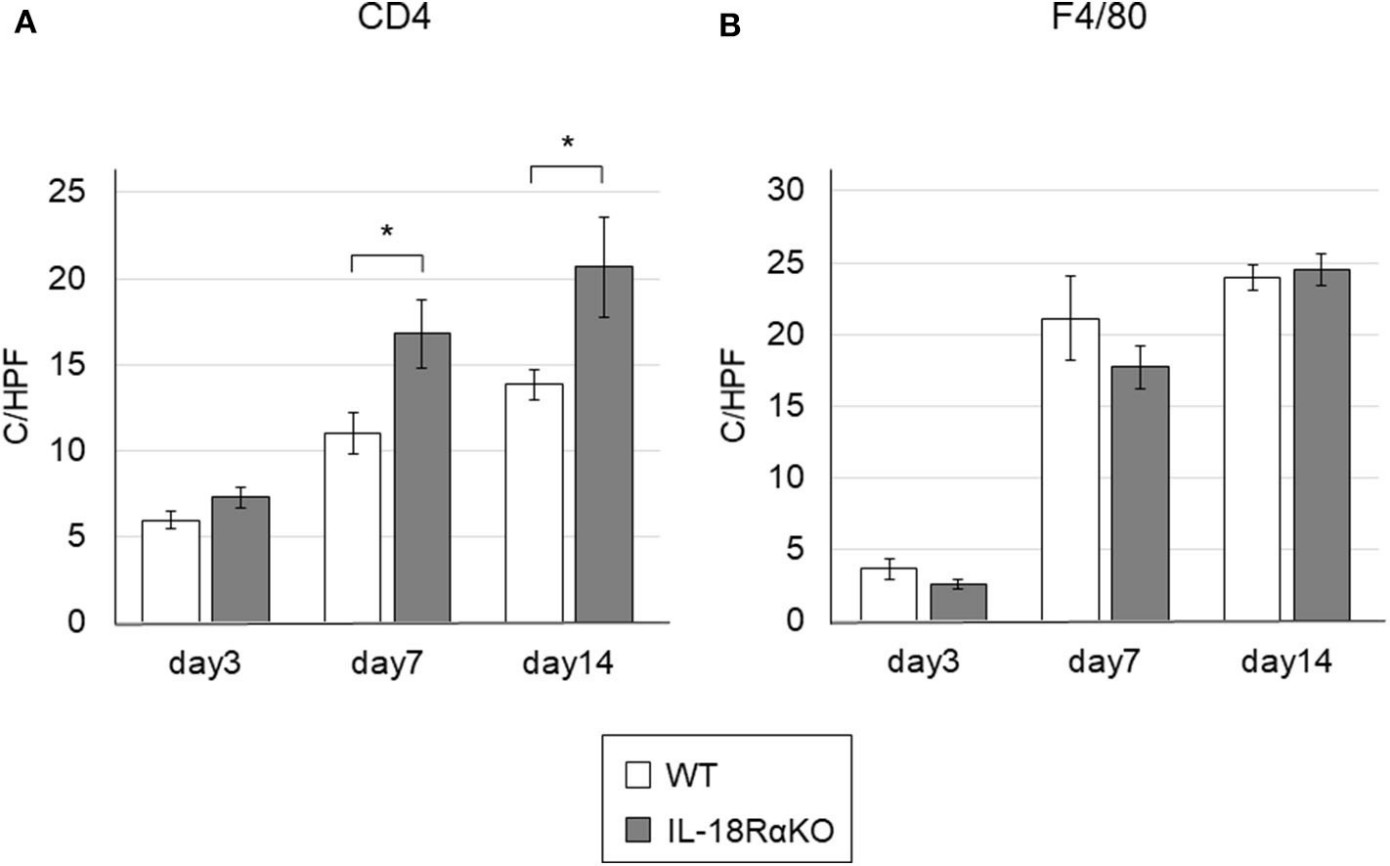
The infiltration of CD4+ T cells and macrophages in the mouse kidney. **(A)** The numbers of interstitial CD4+ cells in IL-18RαKO mice on days 7 and 14 were significantly increased compared to WT mice. **(B)** The numbers of F4/80+ cells as macrophages were not significantly different between WT mice and IL-18RαKO mice. The data are mean ± SEM. *p < 0.05.

We investigated the infiltration of CD4+ T cells and macrophages in the renal interstitium (Figure 7). The number of interstitial CD4+ T cells in IL-18RαKO mice on days 7 and 14 was significantly increased compared to WT mice. The number of F4/80+ macrophages was increased after ureteral ligation. There was no significant difference in macrophage infiltration between WT mice and IL-18RαKO mice.

### Expression of Foxp3

**Figure 8:**
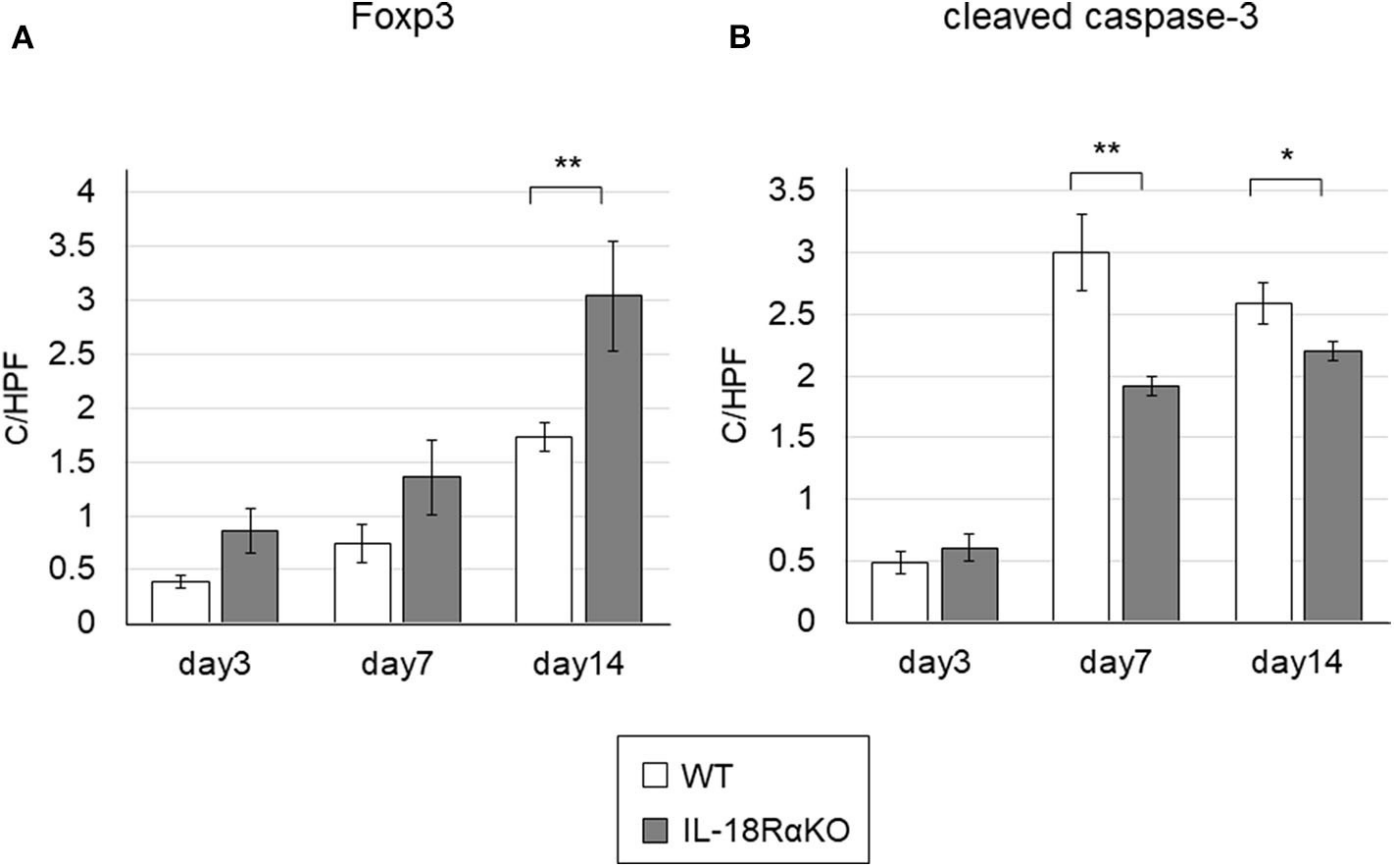
Foxp3+ cells and cleaved caspase-3+ cells in the mouse kidney. **(A)** The number of interstitial Foxp3+ cells in IL-18RαKO mice was increased significantly on day 14 compared to WT mice. **(B)** The numbers of cleaved caspase-3+ tubular cells in IL-18RαKO mice were significantly decreased on days 7 and 14 compared to WT mice. The data are mean ± SEM. *p < 0.05, **p < 0.01.

Figure 8A shows immunohistochemical staining for Foxp3. The number of Foxp3+ cells in IL-18RαKO mice was significantly increased on day 14 compared to WT mice. We also determined the Foxp3 mRNA expression measured by real-time PCR and observed that the expression in IL-18RαKO mice was increased significantly on days 3, 7, and 14 compared to WT mice (Table 3).

### Expression of Cleaved Caspase-3

Examples of the immunohistochemical staining for cleaved caspase-3, a marker of apoptosis, are given in Figure 8B. The number of cleaved caspase-3+ tubular cells was increased after ureteral ligation. On days 7 and 14, a significant reduction in the number of cleaved caspase-3+ tubular cells were present in IL-18RαKO mice compared to WT mice.

### Expression of Renal mRNA

The values of the renal mRNA expressions as measured by real-time PCR are shown in Table 3. There was no significant difference in the mRNA expressions of IFN-γ, TNF-α, TGF-β1, IL-6, IL-10, IL-12, MCP-1, or MMP-2 between WT mice and IL-18RαKO mice.

### Splenocyte Adoptive Transfer Reduced Interstitial Fibrosis

The results of the above experiments suggested that IL-18Rα knockout reduced the renal interstitial fibrosis in the UUO model mice. In the IL-18RαKO kidneys, the number of CD4+ T cells was increased compared to the WT kidneys. Based on these results, in order to determine whether CD4+ T cells affect interstitial fibrosis, we transferred CD4+ T cells extracted from IL-18RαKO mouse splenocytes into WT mice.

**Figure 9:**
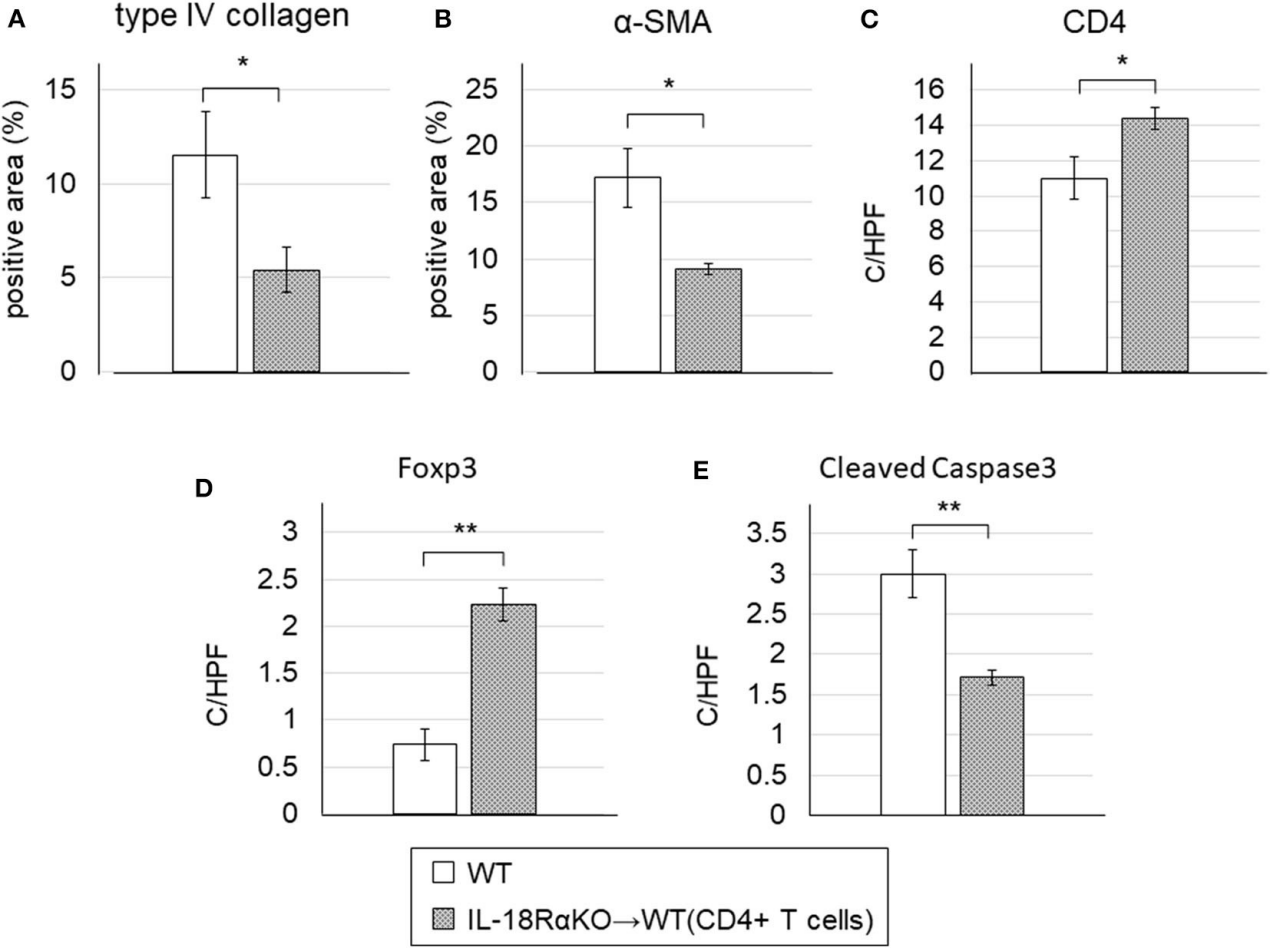
Effects of the adoptive transfer of CD4+ T cells from IL18RαKO mouse splenocytes. CD4+ T cells (2 × 10^6^/mouse) from splenocytes of IL-18RαKO mice were injected intravenously into WT mice 3 days before they mice were subjected to the UUO protocol. WT mice that received a transfer of CD4+ T cells were sacrificed on day 7 after UUO and compared with non-transferred WT mice. WT mice transferred with CD4+ T cells had significantly lower interstitial **(A)** type IV collagen and **(B)** α-SMA expression compared to the non-transferred WT mice. The numbers of **(C)** CD4+ cells and **(D)** Foxp3+ cells were significantly increased and **(E)** the number of cleaved caspase-3+ cells was significantly decreased in the transferred WT mice compared to the non-transferred WT mice. The data are mean ± SEM. *p < 0.05, **p < 0.01.

We then observed that WT mice transferred with CD4+ T cells had significantly lower levels of interstitial type IV collagen and α-SMA expression compared to the WT mice without transferred CD4+ T cells (Figures 9A,B). The numbers of CD4+ T cells and Foxp3+ cells were significantly increased and the number of cleaved caspase-3+ cells was significantly decreased in transferred WT mice compared to non-transferred WT mice (Figures 9C–E).

## Discussion

We have reported that MRL/lpr mice (a well-known model of lupus) cross-bred with mice that are deficient in IL-18Rα exhibited a reduction in autoantibodies, nephritis, and death (16). We also observed that when IL-18RαKO mice experienced a lipopolysaccharide (LPS)-induced acute kidney injury (AKI), they had markedly ameliorated renal function (17). However, a paradoxical finding was obtained in our past studies and other investigations; i.e., that the blockade of IL-18R is associated with an exacerbation of several diseases (14, 18–20). We previously reported that splenic and renal suppressors of cytokine signaling (SOCS)1 and SOCS3 were downregulated in IL-18RαKO mice compared to WT mice with cisplatin-induced acute kidney injury (14). In addition to the inflammatory response, IL-18Rα may induce an anti-inflammatory response by affecting the expression of the cytokine signaling inhibitors SOCS1 and SOCS3. Thus, IL-18/IL-18R interaction may regulate a dynamic balance between destructive and repair signals in renal injury (18). Whether the IL-18/IL-18Rα cytokine signaling pathway acts nephroprotectively may depend on the disease model. In the present study, we investigated whether IL-18R knockout and blockade aggravated or ameliorated renal interstitial fibrosis, and our findings revealed that IL-18RαKO mice had significantly reduced tubular cell apoptosis and significantly suppressed interstitial fibrosis compared to WT mice during obstructive injury. There was no significant difference between WT and IL-18RαKO mice in the results of PAS staining. However, the tubular injury was assessed on a 4-point semi-quantitative scale, which may have obscured the differences in PAS staining between WT and IL-18RαKO mice. We measured BUN levels and found no significant differences between WT and IL-18RαKO mice. This may have been due to compensation by the healthy side of the kidney, which is less likely to reflect renal injury on the obstructed side.

In our previous report using sepsis model mice, we found an increased expression of IL-18 in CD4+ T cells of the spleen (17). Although the origin of IL-18 production was not investigated in this study, a previous report suggests that tubular epithelial cells are the predominant source of IL-18 production during renal occlusion (21). We compared serum IL-18 levels and renal IL-18Rα expression in IL-18RαKO and WT mice. IL-18RαKO mice tended to have lower serum IL-18 levels than WT mice, but the difference was not statistically significant. This may have been due to the small sample size. Brian et al. showed an increase in renal IL-18 levels and IL-18R expression in WT mice compared to IL-18RKO mice after UUO and reported that IL-18 stimulates its own production during renal obstruction via a positive feedback loop involving IL-18R. Based on their findings we consider that inhibition of the IL-18/IL-18Rα signaling pathway by IL-18Rα deficiency blocks intracellular signaling and reduces the expression of cytokines, including IL-18, in the cell nucleus. In our results, renal IL-18Rα expression in IL-18RαKO mice was significantly reduced compared to that in WT mice. Even in the KO model, IL-18Rα KO mice appeared to express low levels of renal IL-18Rα. We analyzed the renal expression of IL-18Rα using the ΔΔCt method. The ΔΔCt method is a comparison of mRNA expression levels between experimental and sham-operated mice, and using this method, we consider that faint levels of expression might be observed as non-specific mRNA expression. The results of this analysis showed that, although IL-18 expression was observed at low levels, it was significantly reduced in IL-18RαKO mice compared to WT mice. Therefore, we do not expect that IL-18 expression affected the conclusions drawn from this study.

Renal fibrosis is believed to be the result of an immune response involving myofibroblast accumulation and matrix deposition (22). TGF-β is a mediator that plays a central role in renal fibrosis, and its inhibition reduces renal fibrosis in animal models (23). Intracellular signal transduction of TGF-β is mediated mainly by smad2/3 phosphorylation, and it acts on ECM accumulation (23). The epithelial mesenchymal transition (EMT) is the mechanism underlying TGF-β-induced renal fibrosis. The tubular EMT is a biological process by which renal tubular cells lose their epithelial phenotype and acquire new mesenchymal features (24). TGF-β plays an important role not only in the induction of the EMT but also in the induction of the endothelial-to-mesenchymal transition (EndMT), and the induction of the EndMT requires an interaction with inflammatory signal transduction pathways such as those involving TNF-α and IL-1β (25). It is also speculated that MCP-1 and MMP-2 contribute to progressive fibrosis (26, 27), whereas IFN-γ is considered to be an antifibrotic cytokine that attenuates renal fibrosis (28, 29).

Herein we investigated various fibrosis-related markers including TGF-β1 by performing a PCR analysis, but we did not obtain any results that could explain the inhibitory effect of IL-18RαKO on renal fibrosis. We also measured TGF-β1 protein levels by western blotting and found no significant difference between WT and IL-18RαKO mice (data not shown). These results suggested that IL-18R is involved in renal interstitial fibrosis by a mechanism that is independent of TNF-α and TGF-β1. Similarly, Bani-Hani et al. reported that transgenic mice with neutralized IL-18 activity exhibited a suppressed EMT and renal fibrosis without demonstrating alterations in TGF-β1 or TNF-α activity (13). Our present findings showed suppression of tubular cell apoptosis in IL-18RαKO mice. The proapoptotic activity of IL-18 has been shown to be mediated through a Fas/FasL-dependent pathway (30).

To elucidate the mechanism of IL-18R-induced renal interstitial fibrosis, we focused on the increase of CD4+ T cells in the kidney of IL-18Rα KO mice. We speculated that Foxp3+ Tregs may play a protective role in tubular cell apoptosis and interstitial fibrosis. We tested these hypotheses by conducting the adoptive transfer of CD4+ T cells from the splenocytes of IL-18Rα KO mice into WT mice, and the results demonstrated the suppression of fibrosis with an increase of Foxp3+ cells in the kidneys of the transferred WT mice. In addition, CD4+ T cells from IL18RαKO mice may have been involved in the induction of Foxp3+ Tregs through a mechanism that remains to be identified.

Tregs are a subset of CD4+ T cells and are characterized by the expression of the Foxp3. Tregs are 5–10% of peripheral CD4+T cells in normal mice and healthy humans, and they play important roles in immune homeostasis and in the suppression of unwanted inflammatory responses to self-antigens (31–33). In animal models, Tregs have been shown to have a renal-protective effect through the suppression of renal inflammation (34–39). In an ischemia-reperfusion injury mouse model, the depletion of Tregs led to worse neutrophil infiltration, tubular damage, and renal function (36, 37). A protective effect of Tregs has also been reported in nephrotoxic renal injury and septic AKI, and Tregs are expected to be the focus of a novel therapeutic approach for these renal diseases (38, 39). Little has been reported on the role of Tregs in obstruction-induced renal interstitial fibrosis via IL-18R.

Our study has some limitations. The cause of the increase in CD4+ T cells in the kidneys of IL-18RαKO mice was not fully examined. In addition, we did not investigate the transcription factors potentially responsible for the differentiation of naive CD4+T cells, such as T-bet, GATA3, and RoRγt, or markers associated with the switch from the Th1 to the Th2 phenotype, such as IL-4, IL5, and Il-13. In addition to CD4+ T cells, IL-18Rα is also expressed on CD8+ T cells and NK cells and is activated by IL-18 (40), but we did not examine the effects of IL-18RαKO on CD8+ T cells and NK cells in the UUO model. Our results did not fully clarify the mechanism by which Tregs are induced in IL-18RαKO mice. In a report showing that IL-12p40 and IL-18 were necessary for viral control and recovery from ectromelia virus infection, Wang et al. reported that splenic Tregs were increased in IL-18-deficient mice (41). IL-6, together with TGF-β, induces the generation of Th17 cells from naive T cells and inhibits Tregs differentiation (42). Thiolat et al. reported that treatment with anti-IL-6 antibodies increased Tregs in the spleens of mice with collagen-induced arthritis (CIA) and the patient sera of rheumatoid arthritis patients (43). In the CIA model, we previously reported that IL-18RαKO mice had reduced IL-6 levels in serum and splenocytes and reduced IFN-γ production in splenic CD4+ T cells compared to the WT mice (44). In the present study, we did not examine the cytokine profile of CD4+ T cells, including IL-6, which may affect Tregs differentiation, in IL-18RαKO mice. Although the reason for the increase in Tregs is not clear, IL-6 may be involved in IL-18Rα-mediated effects on Tregs. Further experiments are required to compare the detailed cytokine profiles of CD4+ T cells in WT and IL-18RαKO mice. In addition, experiments to transplant CD4+ T cells from the bone marrow and kidneys of IL-18RαKO mice into WT mice would be necessary to investigate whether renal or extra-renal IL-18 is more important.

In our present study, although the mechanisms underlying the induction of Tregs and the suppression of fibrosis by Tregs remain unclear, our findings are important in the establishment of the pathology of renal interstitial fibrosis mediated by IL-18 and in the design of therapeutic strategies.

In conclusion, we determined the effect of IL-18RKO in UUO model mice. This study is one of the few that has investigated the relationship between IL-18R and renal interstitial fibrosis. Our results suggest that Foxp3+ Tregs have a protective effect in the pathology of renal interstitial fibrosis via IL-18R. Our findings may provide a new therapeutic target for the control of renal interstitial fibrosis by IL-18.

## Data Availability Statement

The raw data supporting the conclusions of this article are available on request to the corresponding author.

## Ethics Statement

The animal study was reviewed and approved by Kindai University Animal Care Committee Kindai University School of Medicine.

## Author Contributions

YH and YN designed, performed, and interpreted the experiments. YH drafted the manuscript. YH and KN performed the experiments. YH, YN, and AI analyzed the data. YN, MS, KK, MF, and IM edited the manuscript. All authors contributed to the manuscript's revision, read, and approved the submitted version.

## Conflict of Interest

The authors declare that the research was conducted in the absence of any commercial or financial relationships that could be construed as a potential conflict of interest.
